# Prognostic value of the controlling nutritional status score in patients with myelodysplastic syndromes

**DOI:** 10.3389/fnut.2022.902704

**Published:** 2022-07-27

**Authors:** Qiuni Chen, Kankan Chen, Sumei Wang, Lijuan Zhang, Yuye Shi, Shandong Tao, Zhengmei He, Chunling Wang, Liang Yu

**Affiliations:** ^1^Department of Hematology, The Affiliated Huaian No.1 People's Hospital of Nanjing Medical University, Huaian, China; ^2^Key Laboratory of Hematology of Nanjing Medical University, Nanjing, China; ^3^Department of Hematology, The Huai'an Clinical College of Xuzhou Medical University, Huaian, China

**Keywords:** myelodysplastic syndromes (MDS), controlling nutritional status (CONUT), prognosis, nutritional status, retrospective analysis

## Abstract

**Background:**

Myelodysplastic syndromes (MDS) are a heterogeneous spectrum of clonal hematopoietic disorders with varying degrees of cytopenia and morphologic dysplasia. The controlling nutritional status (CONUT) score, an easy-to-use tool for assessing the nutritional status, was reported as an independent prognostic factor in cancer patients. However, its role in patients with MDS is unclear.

**Objective:**

We aimed to explore the impact of CONUT score on the prognosis of patients with MDS, which is of great significance for clinical treatment.

**Methods:**

A total of 121 patients with MDS were analyzed. The CONUT score was calculated prior to therapy. The bio-informatics tool X-tile was used to define the CONUT score and the threshold of 4 points was determined to predict the prognosis. Patients were divided into CONUT^low^ and CONUT^high^ groups, and the characteristics were compared between two groups.

**Results:**

Results show that CONUT^low^ was associated with better overall survival (OS) than CONUT^high^ patients (Median OS, 30.20 vs. 19.63 months, *p* = 0.0003). However, there were no statistical differences in progression-free survival (PFS) between the two groups (*p* = 0.2683). Results of univariate and multivariate COX proportional hazard analysis adjusted for bone marrow blasts level, platelet count, International Prognostic Scoring System (IPSS) scores, gender, and hemoglobin (Hb) level showed that the CONUT score was useful in the evaluation standard of OS of MDS (hazard ratio (*HR*) 2.297, 95% *CI* 1.441–3.663, *p* < 0.001).

**Conclusions:**

The CONUT, as a novel immuno-nutritional biomarker, may be useful in predicting the OS of MDS.

## Introduction

Myelodysplastic syndromes (MDS) are a very heterogeneous group of clonal myeloid neoplasms characterized by the risk of developing acute myeloid leukemia (AML) (1, 2). Treatment of MDS is risk adapted, involving the definition of different goals of therapy according to the risk status of the patient (3). Diagnostics and specific risk stratification are the first steps toward an individual prognostication and treatment. Several prognostic indices such as the International Prognostic Scoring System (IPSS) (4) and Revised IPSS (IPSS-R) (5) have been established and are the most commonly used prognostic models. Nevertheless, the current prognostic staging systems do not consider the role of the patient's nutritional status on the prognosis of MDS.

Nutritional status has been found to be related to the clinical outcomes of patients with cancer (6, 7). In the field of hematologic malignancies, recent studies have suggested that nutritional status is a potential parameter influencing the prognosis of acute leukemia, diffuse large B-cell lymphoma (DLBCL), and multiple myeloma (MM) (8–11). However, the role of nutritional status on the prognosis of MDS is unclear.

The controlling nutritional status (CONUT), calculated based on serum albumin (ALB), total cholesterol concentration (CHO), and total lymphocyte count (ALC), is an easy-to-use and validated tool for assessing the nutritional status of patients (12). Several studies have revealed that the CONUT score is an independent prognostic factor in cancer patients, such as bladder cancer, pancreatic cancer, and hepatocellular carcinoma (13–15). In the field of hematologic malignancies, recent studies have suggested that the CONUT score is a potential parameter influencing the prognosis of acute myeloid leukemia (AML), DLBCL, and MM (9, 16, 17). In the field of DLBCL, Nagata et al. (16) first reported that high CONUT score was associated with poor overall survival (OS) of DLBCL patients. They retrospectively analyzed 476 DLBCL patients and divided them into two groups according to their CONUT scores. The results showed that patients with high CONUT scores (≥4) had poorer 5-year OS (49.0 vs. 83.2%, *p* < 0.001) and 5-year progression-free survival (PFS) (46.1 vs. 73.1%, *p* < 0.001) compared to those with low CONUT scores (<4). The similar conclusions were also found in acute leukemia and MM that high CONUT score indicated poor outcome. However, the role of CONUT on the prognosis of MDS is unclear.

In this study, we retrospectively evaluated the relationship between baseline CONUT Score and clinical outcomes of MDS patients.

## Materials and methods

### Study design and patient selection

Myelodysplastic syndromes are a group of diverse bone marrow disorders in which the bone marrow does not produce enough healthy blood cells. One hundred and sixty-four patients with newly diagnosed MDS between March 2010 and December 2020 were reviewed in the Huai'an No.1 people's hospital. The study population was selected according to the following criteria and followed up to April 2021. This study was carried in accordance with the Helsinki Declaration. All the patients were anonymous. Informed consent was waived because of the retrospective design of the data collection. PFS, primary end point, was defined as the duration from the first treatment to progression of MDS, death of any cause, or the end of clinical follow-up. OS, secondary end point, was defined as the duration from the first treatment to all-cause death or the end of follow-up.

The inclusion criteria: (a) Diagnosed with MDS according to 2008 and 2016 World Health Organization (WHO) definitions; (b) Complete blood samples were approved for experimental analysis; (c) Detailed clinical data were available.

The exclusion criteria: (a) Age <18 years; (b) Incomplete patient information.

### The CONUT scores

The CONUT scores were calculated based on the ALB, CHO, and ALC. Whole-blood samples collected into tubes were used for measurement (Supplementary Table S1). The baseline ALB, CHO, and ALC level at diagnosis was defined as the value that was obtained on the nearest day before the diagnosis. The ALC was measured using XN-9000 (Sysmex, Kobe, Japan). The ALB and CHO were measured using Cobas c 702(Roche, Switzerland). The bio-informatics tool X-tile (12) was used to analyze the CONUT score threshold, and four points was determined as the cut-off point to predict the prognosis. Subjects were classified as CONUT low ( ≤ 4; *N* = 51) or CONUT high (>4; *N* = 70) cohorts.

### Statistical analysis

All analyses were performed with the Statistical Package (SPSS 26.0 Inc., Chicago, Illinois) and Graphpad Prism six (Graphpad Software, CA, USA). Differences of categorical variables were compared by Mann–Whitney *U*-test or chi-Squared test. OS/PFS were estimated adopting Kaplan–Meier method and survival curves were compared by the log-rank test. The X-tile software (Version 3.6.1, Yale University, New Haven, CT, USA) was used to evaluate the optimal cut-off CONUT scores. Univariate and multivariate Cox proportional hazards models were performed to identify the significant prognostic predictors. The hazard ratio (*HR*) and 95% confidence interval (*CI*) were calculated. The significant variables with *p* < 0.05 defined in univariate survival analyses were included for the multivariate analyses to validate the prognostic value of the CONUT. The *p*-value < 0.05 (two-tailed) indicated the statistical significance.

## Results

### Patient characteristics

A total of 121 patients with newly diagnosed MDS were included in the analyses. The characteristics of them were summarized in Table 1. The median age was 65 (59–72) years and 83 (68.5%) were male. Subjects were classified as CONUT^low^ (≤ 4; *N* = 51) and CONUT^high^ (>4; *N* = 70) cohorts. As a closely related to the nutritional characteristics parameters, body mass index (BMI) is also different between two group (*p* = 0.025). The distribution of characteristics such as age, gender, WHO subtype, hemoglobin **(**Hb**)** level, Bone marrow blast count, white blood cell (WBC) count, absolute neutrophil count **(**ANC**)**, platelet (PLT) count, and IPSS subtypes were not significantly different between two groups.

**Table 1:** Characteristics of 121 subjects with myelodysplastic syndromes (MDS).

**Characteristics**	**Total (*****n*** = **121)**	**CONUT score** ≤ **4(*****n*** = **51)**	**CONUT score>4(*****n*** = **70)**	***P***
Age, years, median (IR)	65 (59–72)	65 (60–72)	67.5 (56.3–73.8)	0.452
Male (%)	83 (68.5)	31 (60.7)	52 (74.3)	0.165
WHO subtype (%)				0.180
MDS-SLD	3 (2.5)	0 (0)	3 (4.3)	
MDS-RS-SLD	11 (9.0)	8 (15.7)	3 (4.3)	
MDS-MLD	19 (15.7)	5 (9.8)	14 (20.0)	
MDS-RS-MLD	5 (4.1)	3 (5.9)	2 (2.9)	
MDS-EB1	26 (21.4)	12 (23.5)	14 (20.0)	
MDS-EB2	34 (28.0)	12 (23.5)	22 (31.4)	
MDS with isolated 5q-	2 (1.6)	1 (2.0)	1 (1.4)	
MDS-U	21 (17.3)	10 (19.6)	11 (15.7)	
Hb, g/L, median (IR)	68 (56.5–82)	72 (59.5–83.5)	64.5 (54.3–76.8)	0.063
Bone marrow blast%, median (IR)	4.0 (0.0–8.0)	4.0 (0.5–7.5)	3.0 (0.0–11.8)	0.513
WBC, ×10^9^/L, median (IR)	2.5 (1.6–3.8)	2.3 (1.5–3.4)	2.61 (1.6–4.8)	0.389
ANC, ×10^9^/L, median (IR)	1.1 (0.6–1.9)	1.01 (0.6–1.7)	1.47 (0.7–2.5)	0.100
PLT, ×10^9^/L, median (IR)	45 (20.5–128)	38 (18.5–105)	54 (29.3–165.8)	0.079
IPSS risk group (%)				0.954
Low	8 (6.6)	3 (5.9)	5 (7.1)	
INT-1	70 (57.8)	31 (60.8)	39 (55.7)	
INT-2	30 (24.7)	12 (23.5)	18 (25.7)	
High	13 (10.7)	5 (9.8)	8 (11.4)	
BMI, median (IR)	23.9 (22.0–26.1)	24.21 (22.1–27.0)	23.3 (21.1–25.4)	0.025

ANC, absolute neutrophil count; Hb, hemoglobin; IPSS, International Prognostic Scoring System; IR (InterQuartile Range); MDS, myelodysplastic syndrome; MDS-EB1, MDS with excess blasts-1; MDS-EB2, MDS with excess blasts-2; MDS-MLD, MDS with multilineage dysplasia; MDS-SLD, MDS with single lineage dysplasia; MDS-RS-MLD, MDS with ring sideroblasts with multilineage dysplasia; MDS-RS-SLD, MDS with ring sideroblasts with single lineage dysplasia; MDS-U, MDS unclassifiable; PLT, platelet count; RBC, red blood cell; RDW, red blood cell distribution width; WHO, World Health Organization.

### Association between CONUT scores and clinical outcomes

**Figure 1:**
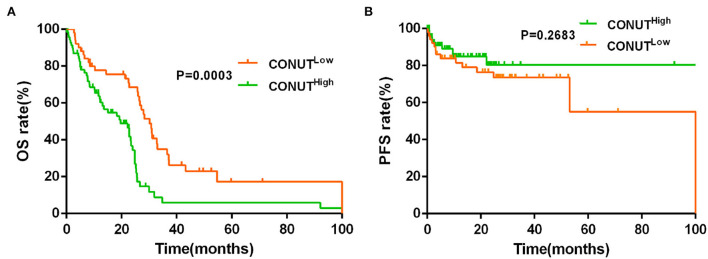
Lower controlling nutritional status (CONUT) was associated with better overall survival (OS) **(A)**, but no with better progression-free survival (PFS) **(B)**.

**Figure 2:**
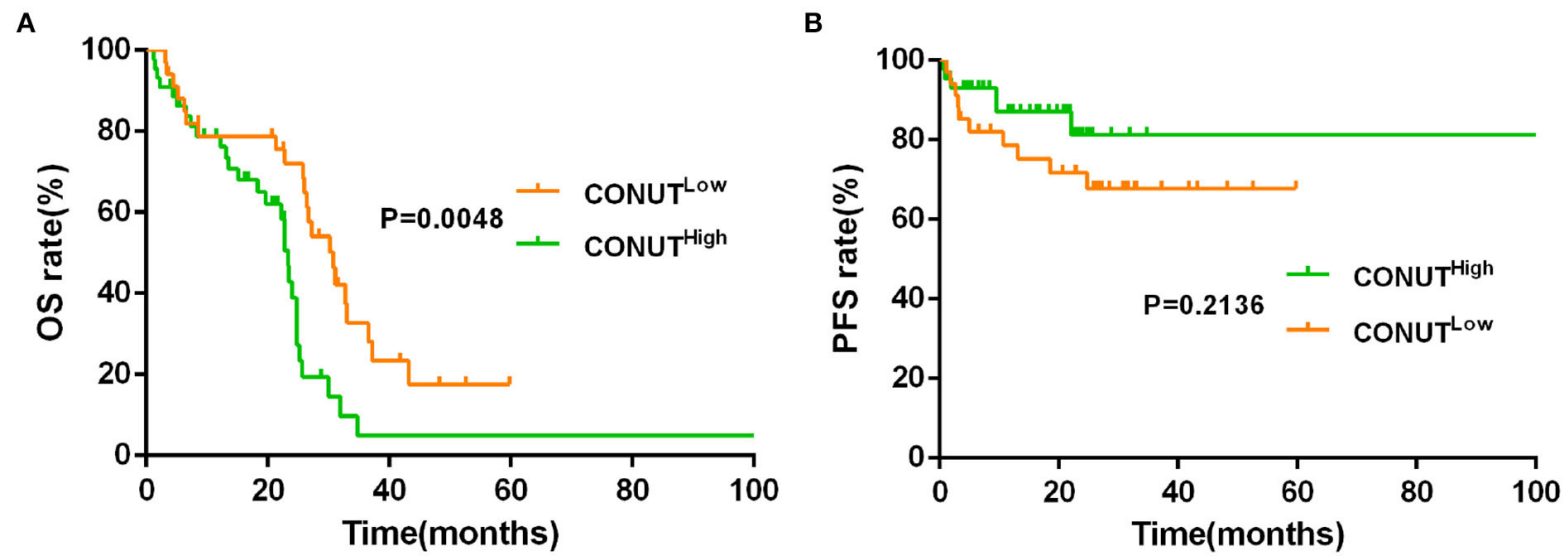
Lower CONUT was associated with better OS **(A)**, but no with better PFS **(B)** in lower risk myelodysplastic syndromes (MDS) groups (lower risk MDS group was defined as International Prognostic Scoring System (IPSS) = low risk + INT-1).

**Figure 3:**
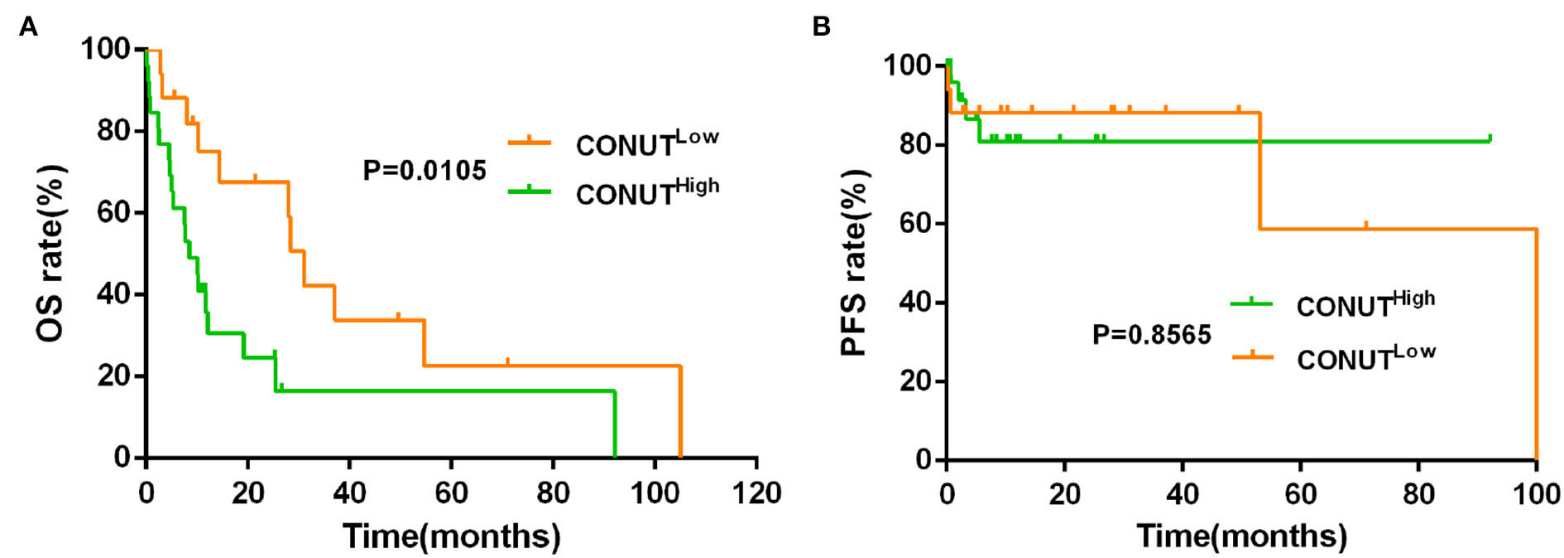
Lower CONUT was associated with better OS **(A)**, but no with better PFS **(B)** in higher risk MDS groups (higher risk MDS group was defined as IPSS = INT-2 + high risk).

The data showed that the CONUT^low^ was associated with better OS compared to CONUT^high^ patients (median OS, 30.20 vs. 19.63 months, *p* = 0.0003, Figure 1A). Although there was a similar tendency in PFS between the two groups, but with no statistically significant difference (*p* = 0.2683, Figure 1B). To determine whether the different risk group (defined by IPSS risk group) will affect the impact of CONUT score on the prognosis of MDS, subgroup analysis was done in lower risk groups (such as low risk and INT-1) and higher risk groups (such as INT-2 and high risk). Patients with lower risk were considered have better prognosis. The results showed that there is a statistical difference (*p* = 0.0048, Figure 2A) in lower risk patients. The same results were found in higher risk subgroups analysis. Patients in higher risk groups who with low CONUT score have better OS compared high CONUT score, respectively (*p* = 0.0105, Figure 3A). We did not find an association of CONUT score with PFS when patients were stratified by the IPSS risk categories (*p* = 0.2136 and *p* = 0.8565, Figures 2B, 3B).

### Univariate and multivariable analyses for OS

Univariate and multivariable analyses were performed to investigate the prognostic factors affecting death (Table 2). In a univariate analysis, CONUT score (*HR* 2.354, 95% *CI* 1.486–3.727, *p* < 0.001), bone marrow blast (*HR* 1.083, 95% *CI* 1.041–1.126, *p* < 0.001), PLT count (*HR* 0.997, 95% *CI* 0.994–1.000, *p* = 0.028), and IPSS scores (*HR* 1.445, 95% *CI* 1.092–1.911, *p* = 0.010) were associated with OS. In a multivariate analysis, parameter with independent adverse significance for the OS was CONUT score (*HR* 2.297, 95% *CI* 1.441–3.663, *p* < 0.001).

**Table 2:** Univariate and multivariable analyses for overall survival (OS).

	**univariate analyses**			**multivariable analyses**		
	**HR**	**95%CI**	***P***	**HR**	**95%CI**	***P***
CONUT score (≤ 4 vs. >4)	2.354	1.486-3.727	<0.001	2.297	1.441-3.663	<0.001
Age (year)	1.016	0.997–1.035	0.090			
Bone marrow blast (%)	1.083	1.041–1.126	0.000	1.077	1.016–1.142	0.013
WBC (×10^9^ /L)	1.042	0.949–1.144	0.388			
ANC (×10^9^ /L)	0.999	0.870–1.147	0.986			
Hb(g/L)	1.008	0.095–1.008	0.095			
PLT (×10^9^ /L)	0.997	0.994–1.000	0.028	0.998	0.996–1.001	0.997
IPSS scores	1.445	1.092–1.911	0.010	0.997	0.660–1.508	0.989
BMI	1.003	0.943–1.066	0.931			

## Discussion

In our study, we performed a retrospective analysis of 121 patients with MDS. The CONUT score was determined prior to therapy. We compared the survival between CONUT^low^ group and CONUT^high^ group and found that high CONUT scores were found to be strongly associated with poor outcome in MDS patients. Further external validations are needed to clarify the accurate prognostic role of the CONUT score for MDS patients.

The previous studies showed that the CONUT score at diagnosis was associated with the prognosis of patients with hematologic malignancies. In the field of MM, Okamoto et al. (17) found that transplant-eligible MM patients with high CONUT scores showed worse OS than those with low scores, and high CONUT score (>4) was an independent prognostic factor in MM patients. In the field of leukemia, Ureshino et al. (18) found that low CONUT score (≤ 3) was correlated with better OS in younger patients with adult T-cell leukemia (ATL). Among 14 younger patients who received allo-HSCT, low CONUT score group had better OS than high group. All those results indicated that the CONUT score could be a prognostic tool for patients with hematologic malignancies. In this study, we found that MDS patients whose CONUT score more than four at diagnosis experienced shorter OS compared to those lower than four. There is seemingly similar tendency in Kaplan–Meier curves of PFS, however, the difference was not significant, maybe due to the small sample sizes and bias in the study.

Univariate and multivariate analyses were also performed to investigate the prognostic factors affecting disease progression and death. Results indicated that CONUT score is a prognostic item in patients with MDS. Although the underline mechanism of action and how high CONUT score indicates true undernutrition or cachexia are still unclear, but it becomes a consensus that CONUT score is a useful tool (19–21), which can be quickly and accurately detected in peripheral blood test at diagnosis.

The CONUT score, as an immuno-nutritional index, is calculated based on ALB, ALC, and CHO. ALB affects not only by nutritional status, but also by liver synthesis capacity (22). It has been shown that ALB synthesis is reduced in many malignancies *via* proinflammatory cytokines such as TNF-a and IL-6 (23). ALC, another component of the CONUT score, is responsible for the cellular immune response of the host (24, 25). Lymphocytes control the host's antitumor activity by inducing apoptosis with their cytotoxic effects in the tumoral microenvironment, which inhibit cancer cell proliferation, invasion, and migration (26, 27). CHO is the main component of the cell membrane. Hypocholesterolemia is associated with the reduction of all lymphocytes, such as CD8 T-cells and suppresses the immune system (28). Each of the three parameters in the CONUT score was reported to reflect cancer prognosis in various types of cancer and there are several reasons for significant prognostic role of the CONUT score in cancer patients (29–31).

Firstly, low ALB was proved to be correlated with poor survival in gastric cancer patients (32). The same conclusions were identified in hematological malignancies, such as MM, AML, and MDS (11, 33). ALB is the most abundant protein in serum and contributes to the maintenance of oncotic pressure as well as to transport of hydrophobic molecules (34). ALB has mainly been considered a biomarker of immunocompetence status and fundamental to nutritional assessment (35). Persistent inflammation contributes to decreased ALB levels (36), plays a central role in the malnutrition, inflammation, which predicts poor clinical outcomes (37). Moreover, as a component of systemic inflammation, ALB is correlated with cancer-related systemic inflammation and tumor progression, which may be caused by the reduced production of ALB by hepatocytes due to the inflammatory cytokines released by the tumor cells (38). Secondly, ALC and absolute monocyte (AMC) counts at the time of treatment initiation were regarded as biomarkers of the tumor microenvironment and immune surveillance in several hematologic malignancies of both myeloid and lymphoid origin (39). Decreasing of ALC counts was shown to be associated with the increasing drug resistance in DLBCL (40). Third, CHO has been found to be related to cancer progression and metastasis, such as colorectal cancer, gallbladder cancer, and MM (41–43). The previous studies have found that lower serum total CHO levels were linked to worse prognosis in renal cell carcinoma and non-small-cell lung cancer (44, 45). It is not clear whether tumor progression causes hypocholesterolemia or hypocholesterolemia triggers tumor progression. Increased levels of cytokines such as IL-6 and activation of NF-kB associated with increased inflammation because of proliferation of tumor cells can cause hypocholesterolemia (46). There was no research exploring the role of CHO on the prognosis in patients with MDS.

Accordingly, the CONUT score as a combination of these three factors that might be a better prognostic factor for MDS. The status of nutrition is associated with the treatment response and the therapy-related side effects and toxicity, which also are factors associated with the prognosis of diseases. Therefore, early nutritional status screening and assessment may be important to provide comprehensive algorithms for individualized clinical treatment in cancer patients. As an easy get index in hospital, CONUT score can assess the nutritional status of patients more comprehensively.

There are some limitations in this study. First, as in most retrospective analyses, selection bias in collection cannot be completely avoided. Second, we could not find the significant correlation between the type of therapy and the survival, which may because of substandard treatment and small sample size.

## Data availability statement

The original contributions presented in the study are included in the article/Supplementary materials, further inquiries can be directed to the corresponding author/s.

## Ethics statement

The studies involving human participants were reviewed and approved by Institutional Review Board of the Affiliated Huaian No.1 People's Hospital of Nanjing Medical University. Written informed consent for participation was not required for this study in accordance with the national legislation and the institutional requirements.

## Author contributions

LY designed the study. QC and KC wrote the manuscript. SW collected data. LZ was responsible for the tables. ST and ZH were responsible for the figures. CW and LY modified the manuscript. All authors contributed to the article and approved the submitted version.

## Funding

This work was funded by the Science and Technology Fund of Huaian City [grant # HAB202020] and Commission of Health of Jiangsu Province [grant # 2019082].

## Conflict of interest

The authors declare that the research was conducted in the absence of any commercial or financial relationships that could be construed as a potential conflict of interest.

## Publisher's note

All claims expressed in this article are solely those of the authors and do not necessarily represent those of their affiliated organizations, or those of the publisher, the editors and the reviewers. Any product that may be evaluated in this article, or claim that may be made by its manufacturer, is not guaranteed or endorsed by the publisher.
