# Supplementary material for: Prognostic value of the controlling nutritional status score in patients with myelodysplastic syndromes
Source: Front Nutr. 2022 Jul 27;9:902704. doi: 10.3389/fnut.2022.902704 (PMC9363897; doi:10.3389/fnut.2022.902704)
Supplement: Supplementary file 1 [file Table_1.DOCX]

**Table S1** Scoring criteria for CONUT score

| Factors | Normal | Mild | Moderate | Severe |
| --- | --- | --- | --- | --- |
| ALB (g/L) | ≥35.0 | 34.9-30.0 | 29.9-25.0 | <25.0 |
| Score | 0 | 2 | 4 | 6 |
| ALC(/L) | >1.6 | 1.2-1.6 | 0.8-1.2 | <0.8 |
| Score | 0 | 1 | 2 | 3 |
| CHO (mg/L) | ≥18.0 | 14.0-17.9 | 10.0-13.9 | <10.0 |
| Score | 0 | 1 | 2 | 3 |
| Total score | 0-1 | 2-4 | 5-8 | 9-12 |
